# Diagnostic Pathology in 2011: reflecting on the development of an open access journal during the last five years

**DOI:** 10.1186/1746-1596-6-129

**Published:** 2011-12-30

**Authors:** Klaus Kayser

## 

Today, electronic communication is involved in all parts of our lives, either in a directed active, communicative, or passive manner. Whether to live with electronic communication or to ignore it, is no longer a question. Instead, the question we have to answer is: How shall we live in our communicative environment? What can we expect? What are we forced to develop in order "to survive"?

This Editorial written at the end of a really successful year of our journal *Diagnostic Pathology *tries to give some answers from different points of view.

Let us start with the publisher's interest which stands on two feet, the commercial success and the scientific reputation. Both feet are linked: The higher the scientific reputation of the journal, the higher the financial benefit, in terms of profit per article, as more articles are likely to be submitted as the reputation increases. The limitations of the required fee are the absolute numbers of published articles: If the publication procedure has not been outsourced the publication expenses are related to a fixed amount that is mandatory for hardware, software, salaries, etc., and upon a relative amount which can be calculated to the number of published articles (manpower required for each article). Thus, publishing more and more articles per year usually will, however may not always, increase the publisher's financial profit. Publishing a low number of articles with a very high reputation might endanger the journal's existence. At present, our journal *Diagnostic Pathology *is in a good shape: in 2011 we will publish more than 100 articles. The citation index has slowly reached 1.39. The percentage of research articles has increased to 60% - 70%, whereas the percentage of case reports has decreased to about 20%. This situation is in favour of a rising citation index despite the negative influence of doubling the published articles.

Naturally, the authors' point of view is somewhat different to the publisher's interest. The publication fee is not small, and can usually only be covered by specific grants. The granting of waivers can only partly solve the "Tom Sawyer's problem" which is searching for outstanding articles and afterwards asking for money to cover their publication. This conflict can only be solved by the journal's attraction to its readers, through its formal work and presented content. Only if these parameters can overcome the constraint of the publication fee, will *Diagnostic Pathology *survive. The steep increase of submissions and published articles seems to confirm that our journal *Diagnostic Pathology *is on a successful way.

The main aim of any Editor-in-Chief is to guide the scientific journal to higher levels of its scientific reputation. Herein both the citation index with all its questionnaires and the geographical distribution of the journal are the main components. The open access journal *Diagnostic Pathology *started six years ago. It possesses more than 1200 registered readers living in all parts of the world. Its scientific reputation lies in the middle of all Thomson Reuter's registered scientific journals. The number of submissions and publications doubled last year and our rejection rate is currently about 50%. So far this data can be compared to other journals, i.e. the more traditional print based, or other open access journals. What else?

We have been involved in electronic publication since its beginning in the early 1990s, starting with the solely electronically distributed Electronic Journal of Pathology and Histology. Since that time new ideas and trials in scientific publication were also in our focus. The first trials of interactive publications were undertaken as well as publication of articles that included executable programs. The reader could start and execute these programs by selecting specific parameters or functions without influence from the authors. Our philosophy to continue to develop electronic scientific publication has remained unchanged since then. We are proud to offer our authors the possibility to publish whole digitized glass slides (virtual slides, VS) without any additional costs since January 2011. To our knowledge, *Diagnostic Pathology *is the first and until today the only, electronically scientific journal that offers its authors the unique chance to publish whole VS, and not only specific areas of interest, in the form of still images. This opportunity has been accepted by about 50% of authors with suitable articles. It took some time to solve the difficult logistic problems but the gateway is now open, and additional derived innovative offers will follow. One of these is that VS and their corresponding articles will be collected to form a repository that can be used for education, as a basis for additional secondary publications (interactive publication), and for assistance in routine diagnostic work. It will also serve as quality assurance for newly submitted articles.

An additional approach will be the "opening" of published articles for the inclusion of related data submitted by other authors. We are also undertaking tests on how to include the reader into published articles, and some of the results from these tests so far are really promising. Of course, the original authors have to be informed and have to give their permission for such investigations.

Open access journals can provide the framework that is mandatory for such new publication and communication techniques. Additional trials would be to automatically include published articles into specific social forums, for example facebook or linkedin. Certainly, we are also thinking about how to improve the review process, as one of our main aims is to judge all submitted articles without personal biased methods that are only related to scientific issues.

*Diagnostic Pathology *is an alive scientific journal. All active journals require certain changes and exchanges from time to time. Unfortunately, one of our active Section Editors, Professor Torsten Goldmann, PhD will be leaving, and we want to express our gratitude for all of his efforts in helping to develop our journal *Diagnostic Pathology*. Similarly, some colleagues of our Editorial board will leave in a routine exchange. We would like to thank all of them for being on the board and supporting the journal. We also regret to announce that one of our internationally well known Editorial board members, Professor Dr. Anthony Leong, has passed away. Certainly, we will keep him in good memory.

We are looking forward to further developing our open access journal *Diagnostic Pathology*, focusing on still not, or only poorly investigated, fields of electronic scientific communication. We will also keep an eye on the progress of molecular and genetic pathology, and on new technologies such as in vivo biopsies, alive imaging publication, or nanotechnologies. For all these promising and growing fields of pathology we do need and appreciate the interest, knowledge, information, and assistance of the scientific community, specifically from our readers and authors.

For the progress made so far we would like to express our deep gratitude to our authors, readers, reviewers, and our publication team, and we wish them all a Blessed and Merry Christmas and a Happy, Healthy, and Fruitful New Year.

Klaus Kayser

Editor-in-Chief

